# Supplementary figures and images for: Expression of transgenic biotin ligases in inducible neuronal murine cell lines by integration into the mHipp11 gene locus
Source: PLoS One. 2025 Mar 4;20(3):e0315806. doi: 10.1371/journal.pone.0315806 (PMC11878913; doi:10.1371/journal.pone.0315806)

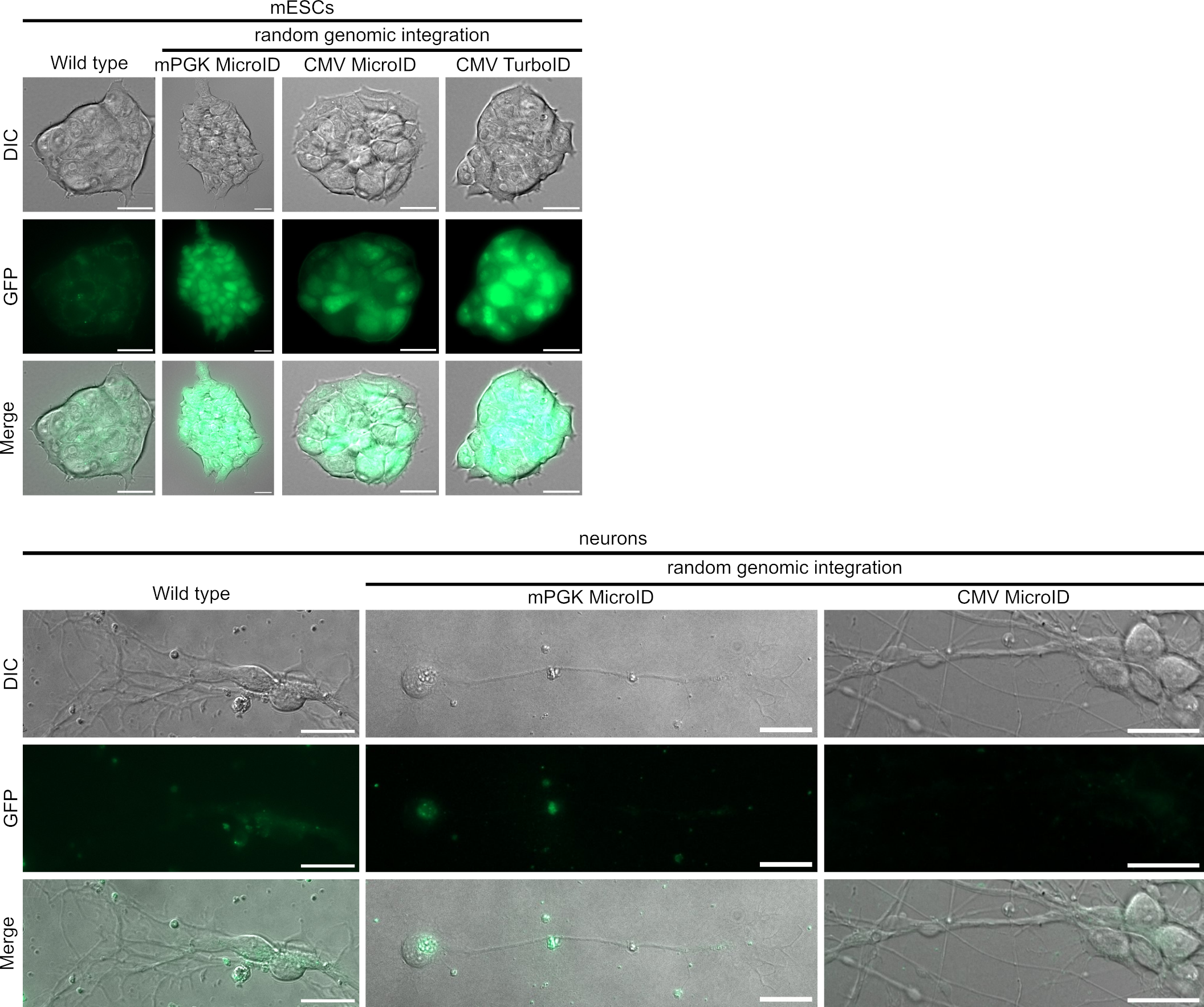

Supplement: S1 Fig — The expression construct was randomly integrated into the genome. Only background signal is detected in neurons, whereas a strong nuclear eGFP signal in mESCs is present. Scale bars: 20 µm. (TIFF) [file pone.0315806.s001.tiff]

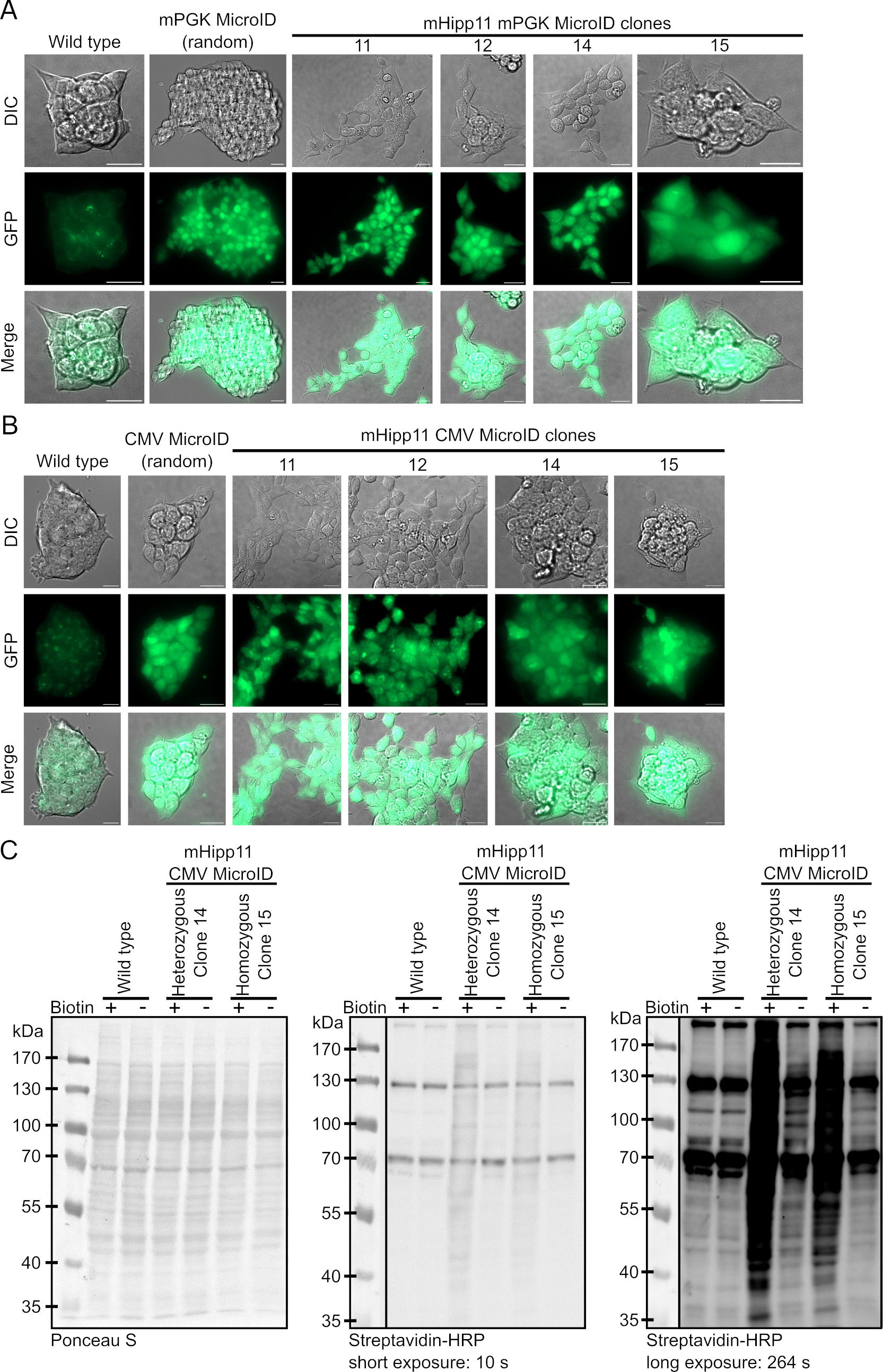

Supplement: S2 Fig — (A) Live imaging of 2xMCP-eGFP-MicroID expressed from a mPGK promoter after random integration into the mESC genome (second column) or targeted integration into the mHipp11 locus (four different clones shown). (B) Live imaging of 2xMCP-eGFP-MicroID expressed from a CMV promoter after random integration into the mESC genome (second column) or targeted integration into the mHipp11 locus (four different clones shown). (C) Biotinylation activity of two clones expressing 2xMCP-eGFP-MicroID from the mHipp11 locus. Biotinylated proteins are detected via streptavidin-HRP. Two different exposures of the same blot are shown. Strong biotinylation is only detectable in MicroID clones after addition of excess biotin. Scale bar: 20 µm. (TIFF) [file pone.0315806.s002.tiff]

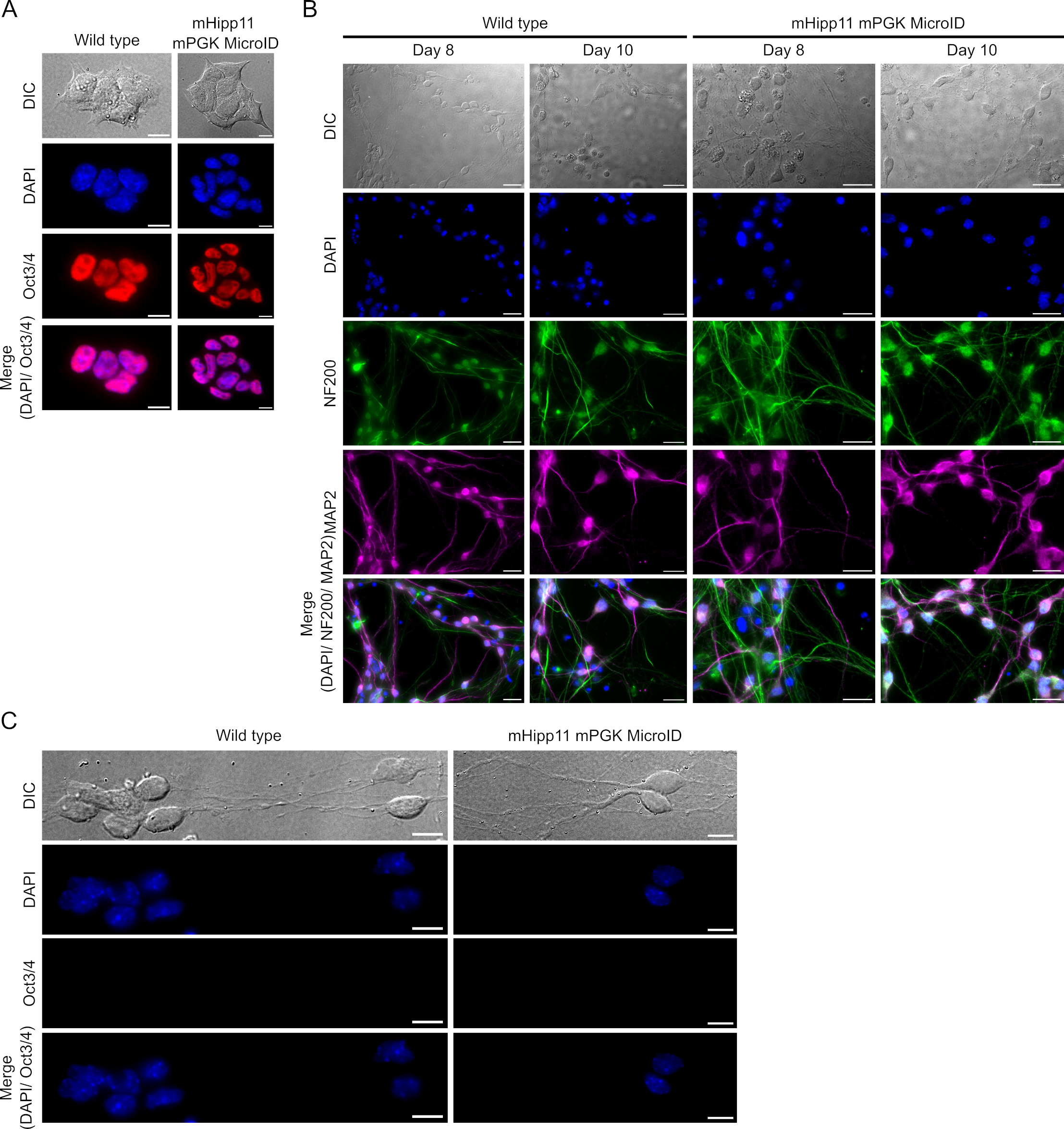

Supplement: S3 Fig — (A) Wild type and mHipp11 mPGK MicroID mESCs were stained with an anti-Oct3/4 antibody to validate pluripotency. Merged images show overlapping DAPI and Oct3/4 signal in the nucleus. Scale bars: 10 µm. (B) Wild type and mHipp11 mPGK MicroID mESC-derived neurons (clone 15) were differentiated until day 8 and day 10 and stained with anti-NF200 antibody and anti-MAP2 antibody to validate neuronal markers. Merged images are composites of DAPI, NF200 and MAP2 staining. Scale bar: 20 µm. (C) Wild type and mHipp11 mPGK MicroID mESC-derived neurons were stained with an anti-Oct3/4 antibody to verify loss of pluripotency after differentiation. (TIFF) [file pone.0315806.s003.tiff]

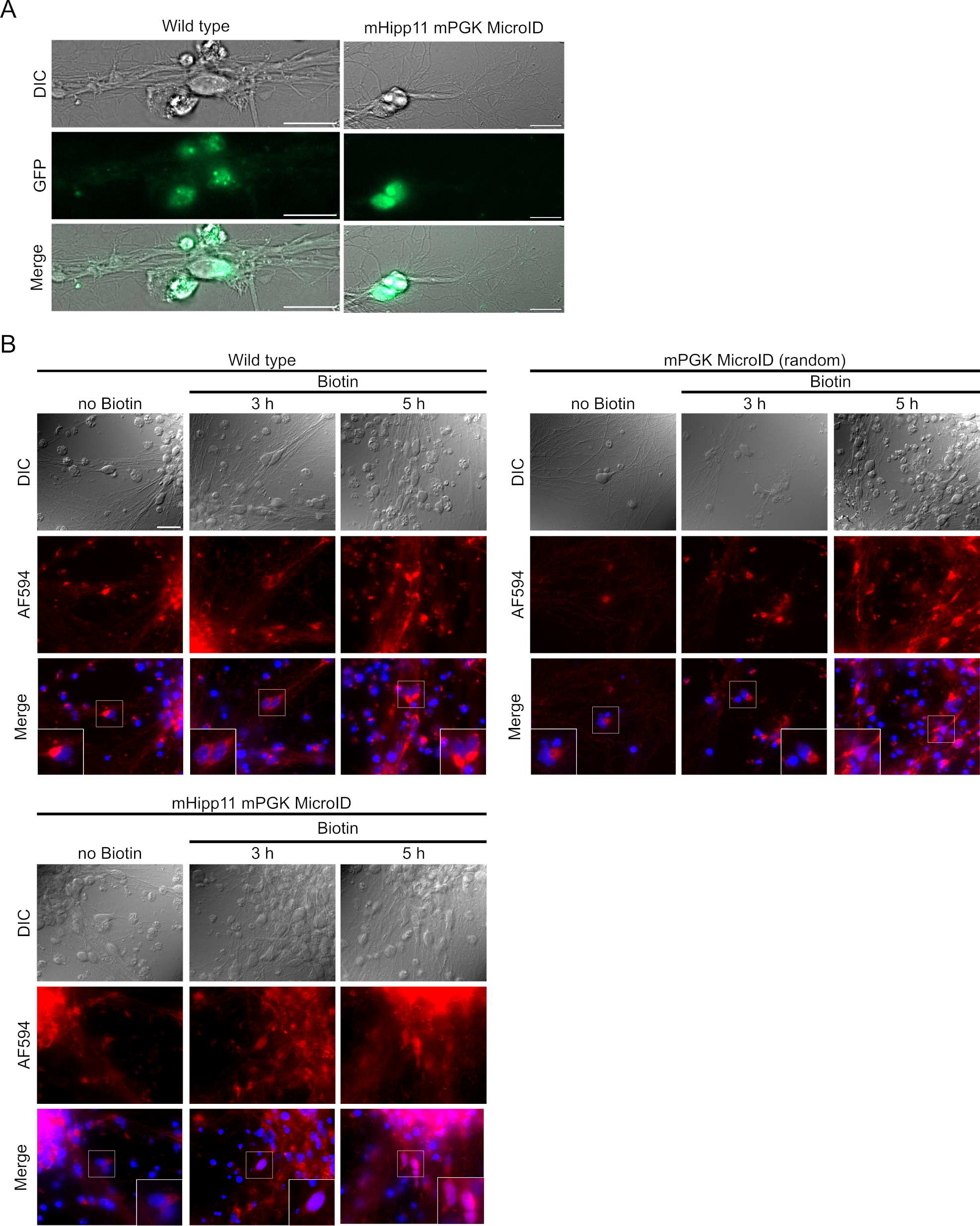

Supplement: S4 Fig — (A) MicroID construct expression in mESC-derived neurons was confirmed by live-cell imaging. Wild type cells show no nuclear eGFP signal. (B) Biotinylation activity in mESC-derived neurons at different time points was visualized by streptavidin-coupled Alexa Fluor 594. Scale bars: 20 µm. (TIFF) [file pone.0315806.s004.tiff]

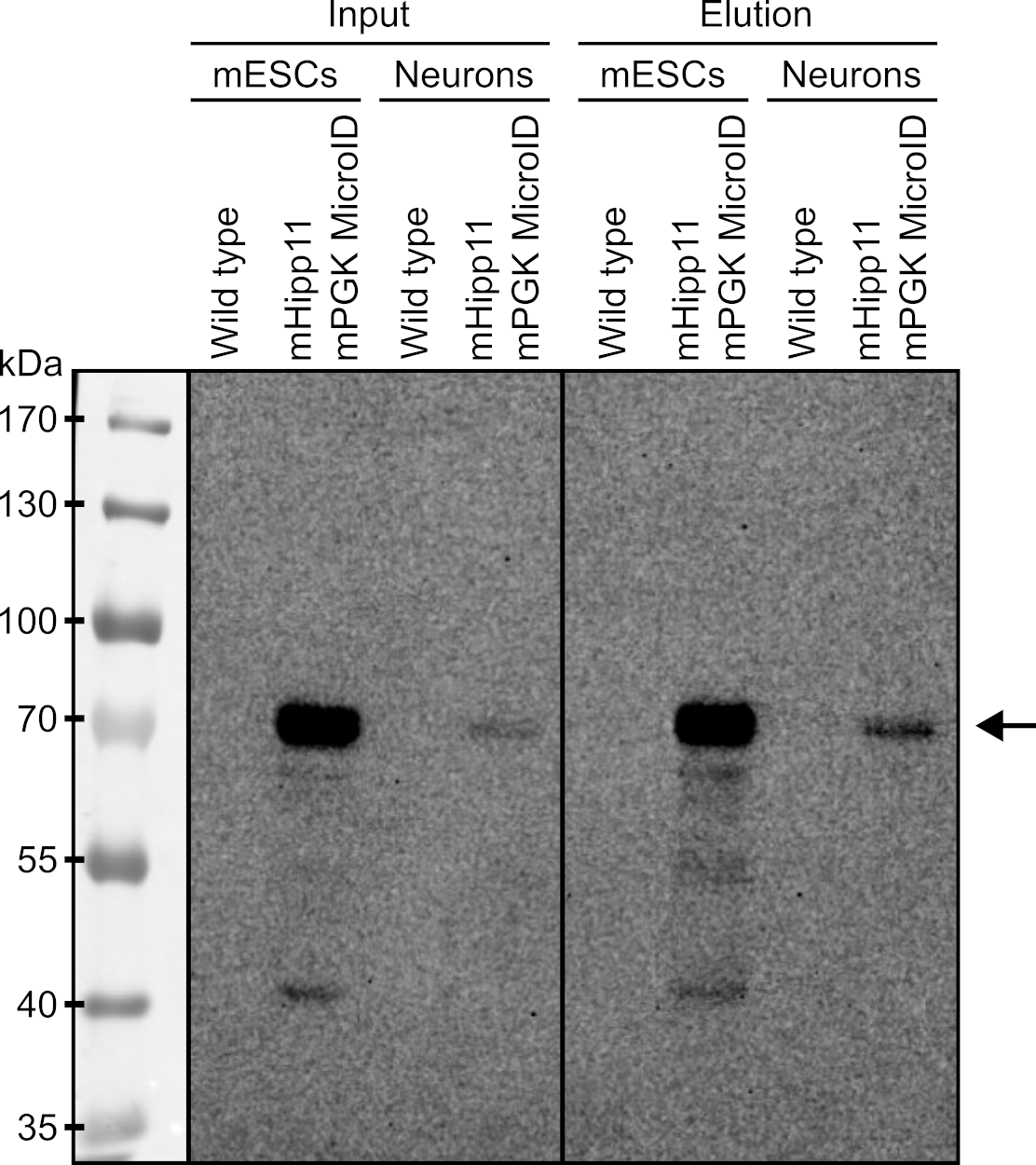

Supplement: S5 Fig — MicroID fusion protein (70 kDa, arrow) was detected by an anti-GFP antibody in total cell lysates (left lanes) and after enrichment by pulldown (right lanes). (TIFF) [file pone.0315806.s005.tiff]
