# Supplementary material for: Expression of transgenic biotin ligases in inducible neuronal murine cell lines by integration into the mHipp11 gene locus
Source: PLoS One. 2025 Mar 4;20(3):e0315806. doi: 10.1371/journal.pone.0315806 (PMC11878913; doi:10.1371/journal.pone.0315806)
Supplement: S6 Fig — (PDF) [file pone.0315806.s006.pdf]

Fig. 1B: upper panel

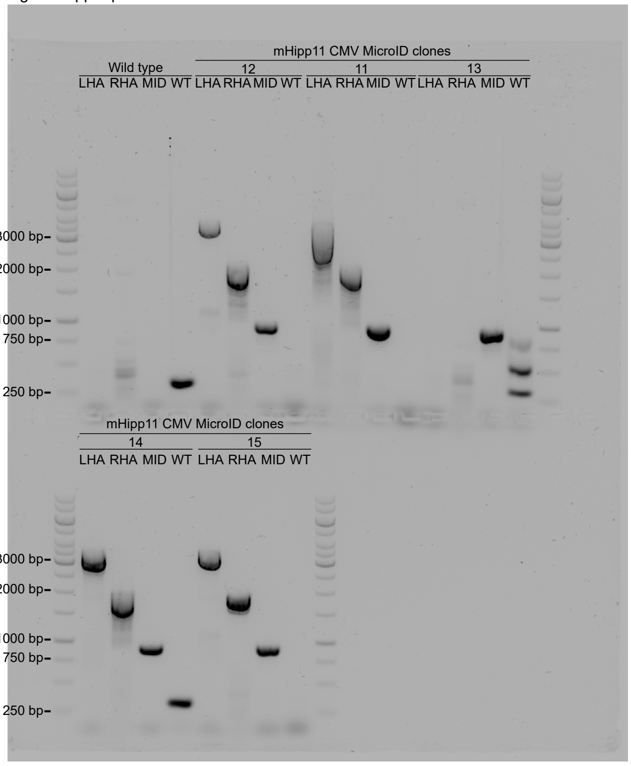

Gel Red dye: 0.151 s exposure time (UV)

Fig. 1B: lower panel

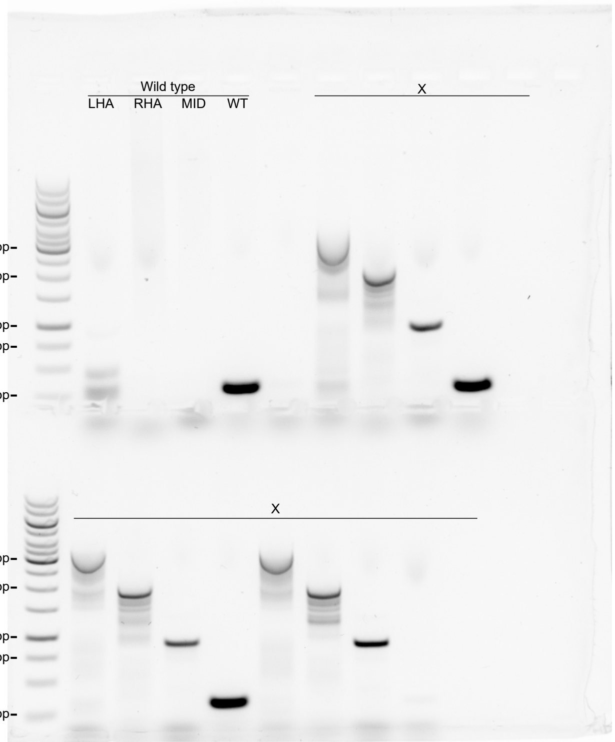

Gel Red dye: 0.122 s exposure time (UV)

Fig. 1B: lower panel

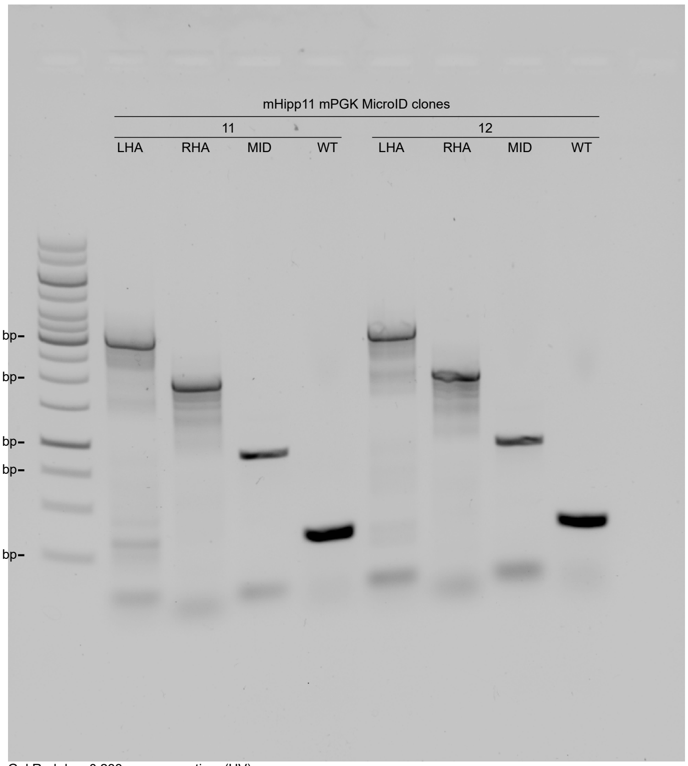

Gel Red dye: 0.233 s exposure time (UV)

Fig. 1B: lower panel

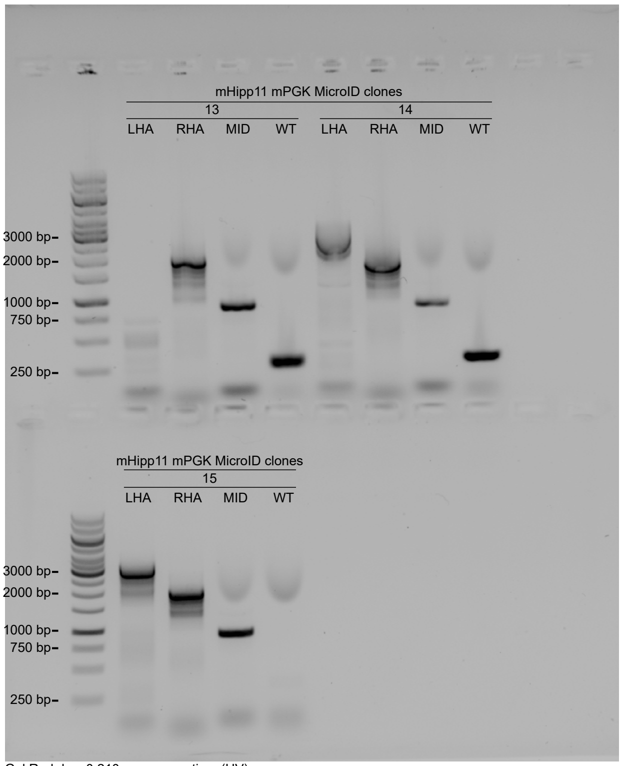

Gel Red dye: 0.210 s exposure time (UV)

Fig. 2B:

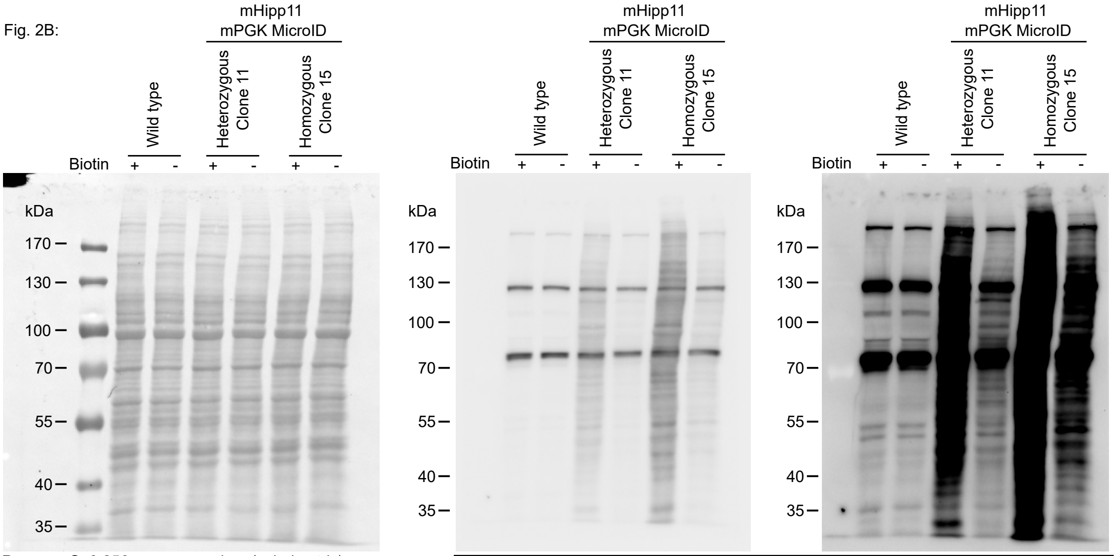

Ponceau S: 0.256 s exposure time (colorimetric)

Streptavidin-HRP (Chemiluminescence): SAM imaging
Left: 10 s exposure time; Right: 200.713 s exposure time

Fig. 3B:

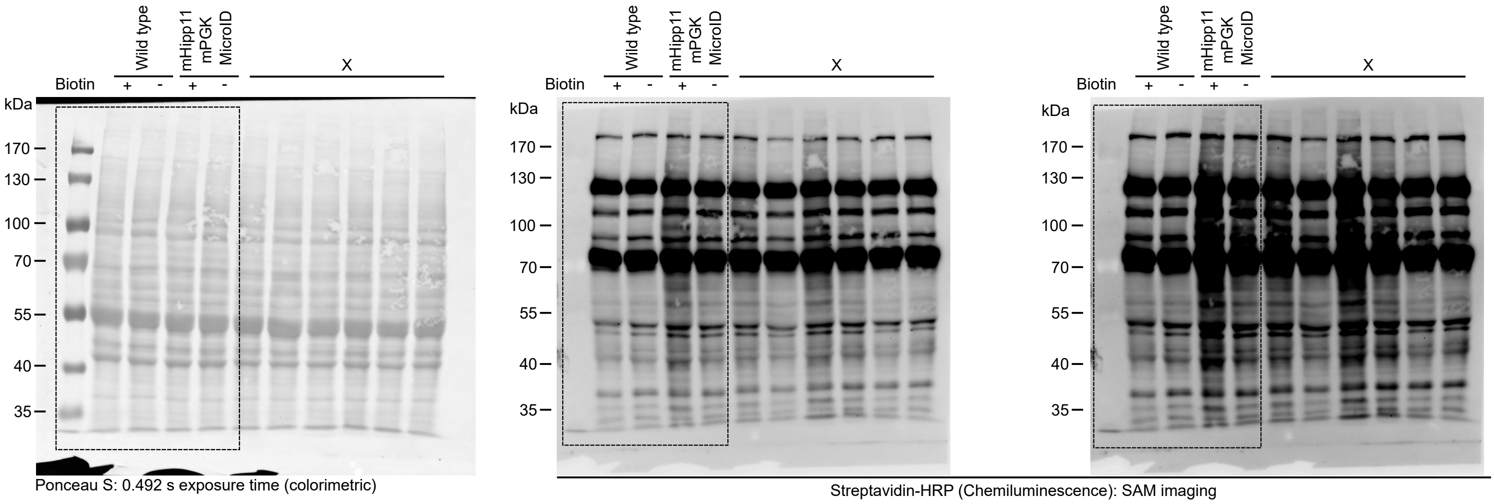

Ponceau S: 0.492 s exposure time (colorimetric)

Streptavidin-HRP (Chemiluminescence): SAM imaging
Left: 137.142 s exposure time; Right: 264.284 s exposure time

Fig. 4A:

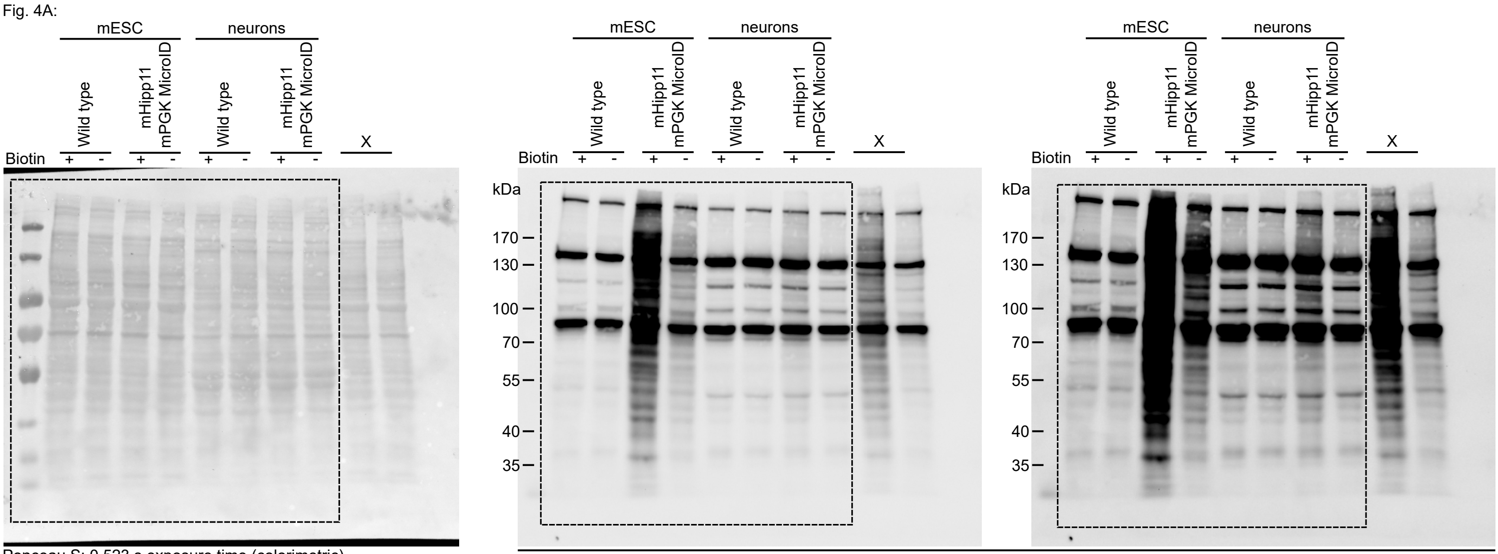

Ponceau S: 0.523 s exposure time (colorimetric)

Streptavidin-HRP (Chemiluminescence): SAM imaging
Left: 72 s exposure time; Right: 269 s exposure time

Fig 4C:

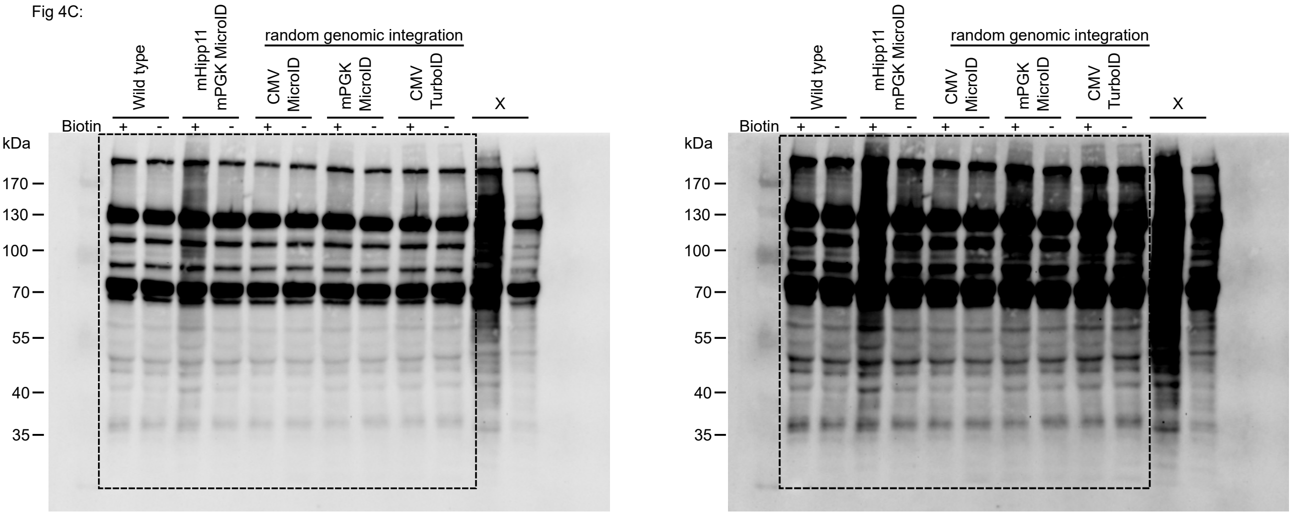

Streptavidin-HRP (Chemiluminescence): SAM images, Left: 73.571 s exposure time; Right: 264.284 s exposure time

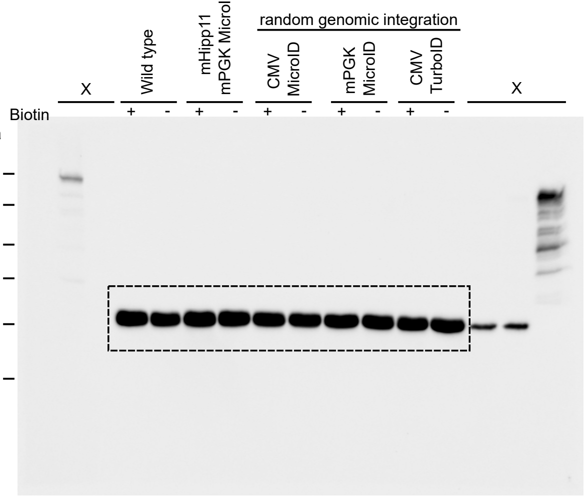

α Tubulin + mouse-hrp (Chemiluminescence): SAM imaging, 10 s exposure time

S2 Fig:

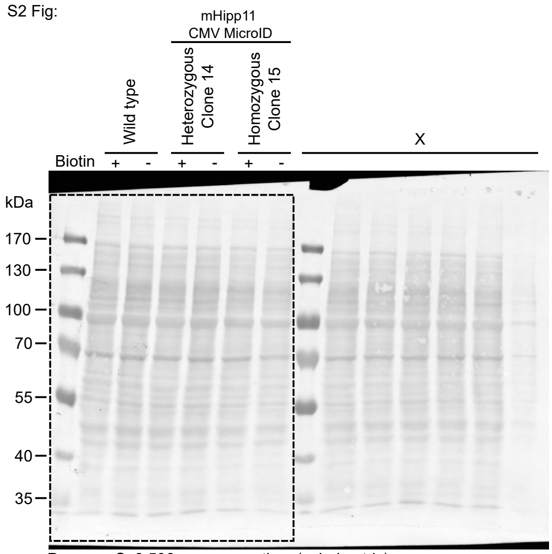

Ponceau S: 0.506 s exposure time (colorimetric)

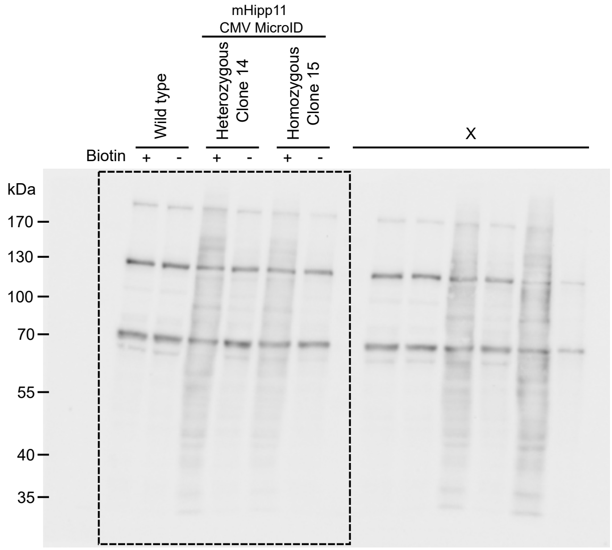

Streptavidin-HRP (Chemiluminescence): SAM imaging, 10 s exposure time

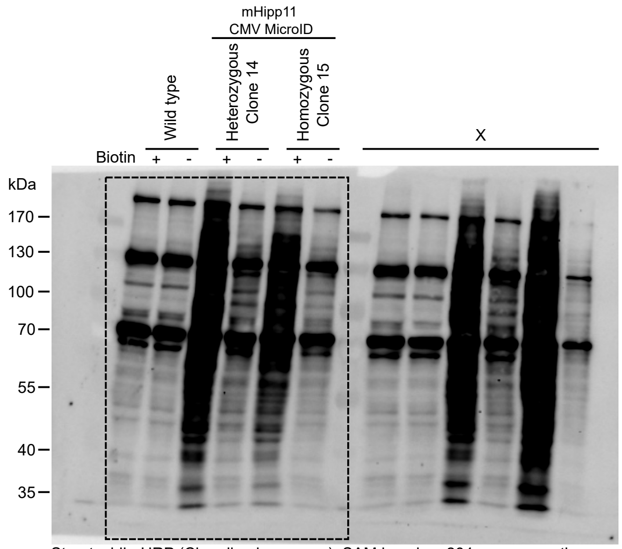

Streptavidin-HRP (Chemiluminescence): SAM imaging, 264 s exposure time

S5 Fig:

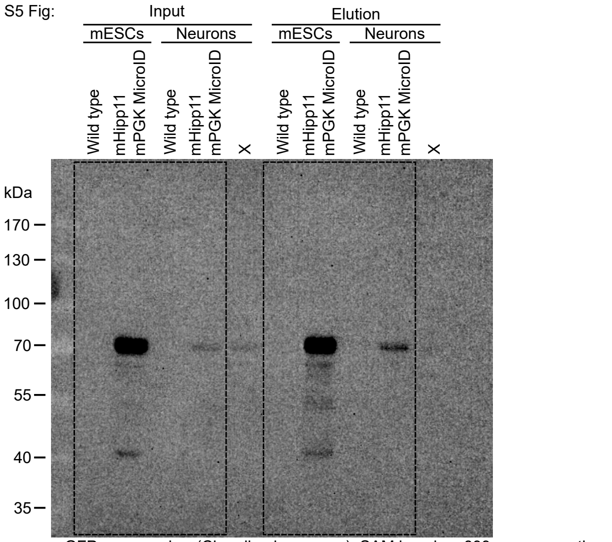

α GFP + mouse-hrp (Chemiluminescence): SAM imaging, 899 s exposure time

Fig. 2B:

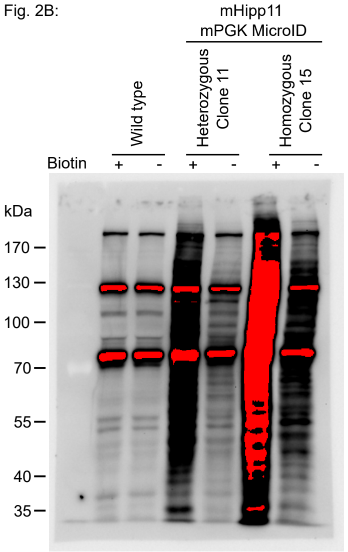

Streptavidin-HRP (Chemiluminescence):
SAM imaging, 200.713 s exposure time

Fig. 3B:

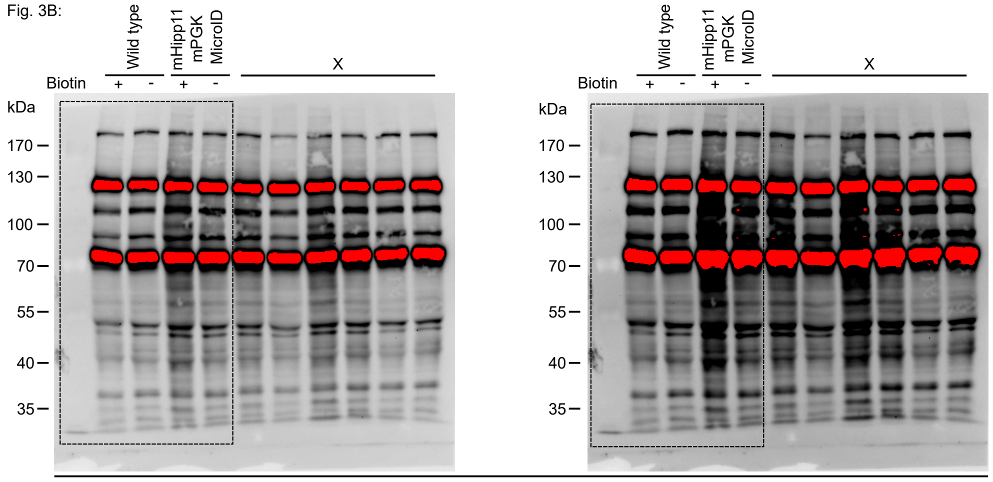

Streptavidin-HRP (Chemiluminescence): SAM imaging
Left: 137.142 s exposure time; Right: 264.284 s exposure time

Fig. 4A:

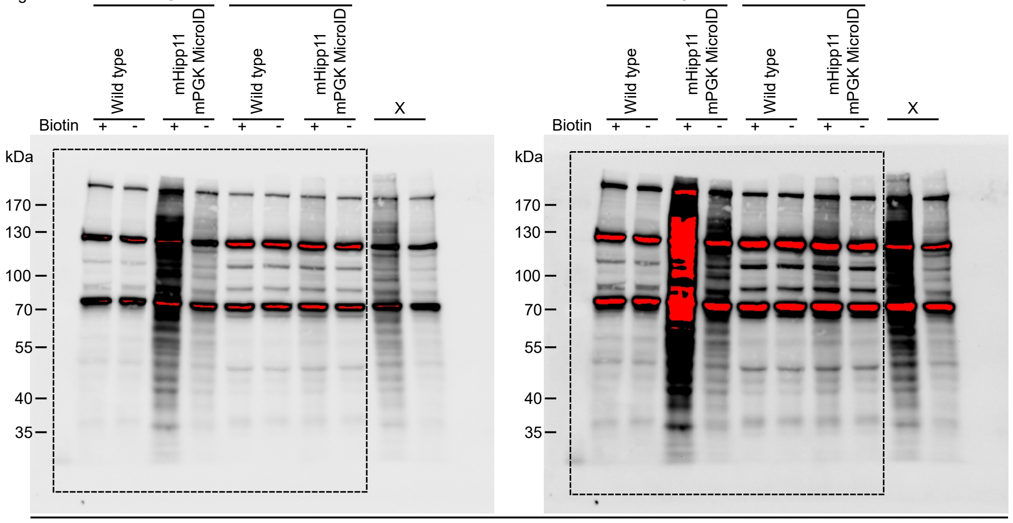

Streptavidin-HRP (Chemiluminescence): SAM imaging
Left: 72 s exposure time; Right: 269 s exposure time

Fig 4C:

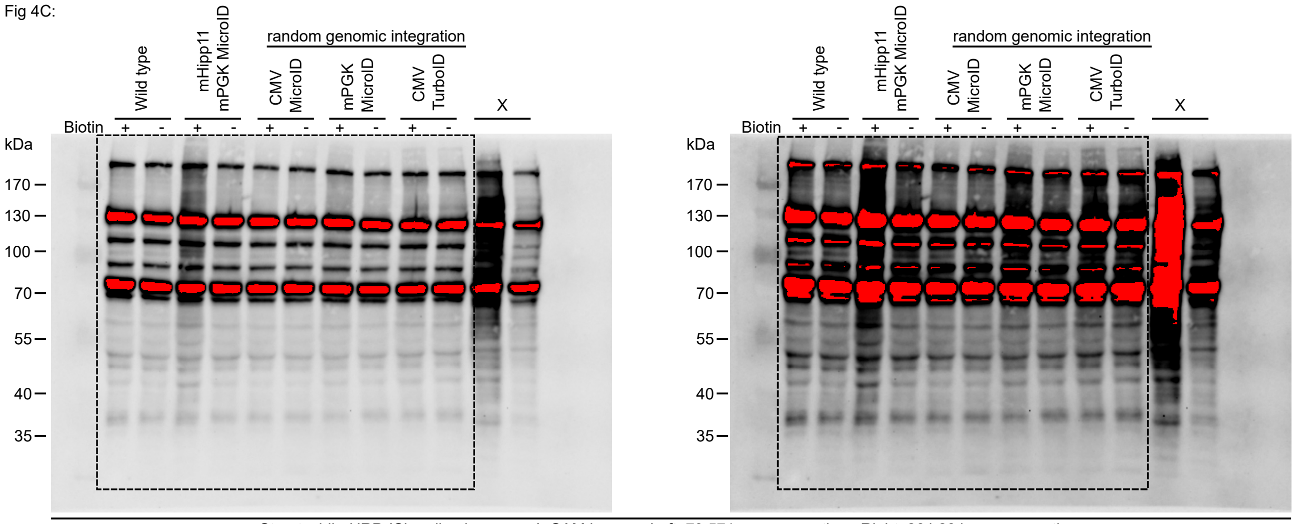

Streptavidin-HRP (Chemiluminescence): SAM images, Left: 73.571 s exposure time; Right: 264.284 s exposure time

S2 Fig:

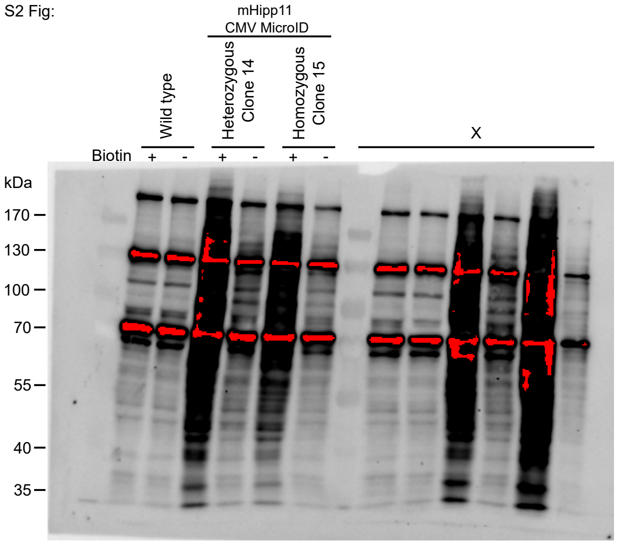

Streptavidin-HRP (Chemiluminescence): SAM imaging, 264 s exposure time

Blots are identical to the ones on previous pages
but with saturated areas displayed in red color.
