# Supplementary material for: Expression of transgenic biotin ligases in inducible neuronal murine cell lines by integration into the mHipp11 gene locus
Source: PLoS One. 2025 Mar 4;20(3):e0315806. doi: 10.1371/journal.pone.0315806 (PMC11878913; doi:10.1371/journal.pone.0315806)
Supplement: S1 Table — (PDF) [file pone.0315806.s007.pdf]

S1 Table. Primers used in this study.

ID	Name	Sequence	Used for
#7040	MicroID pcDNA3.1 F	CACTCTCGGCATGGACGAGCTGTACAAGC TCGAGTTCAAGAACCTGATCTGGCTG	Cloning
#7041	MicroID pcDNA3.1 R	AACGGGCCCTCTAGACTCGAGCGGCCGCT TACTTCTCCTTGAACTTCTTCAGG	Cloning
#7349	MluI XbaI PGK_fwd	TAAGCAACGCGTTCTAGAGGGTAGGGGAG GCG	Cloning
#7353	AflII PGK_rev	TCGTTACTTAAGCGAAAGGCCCGGAGATG AG	Cloning
#7377	SLIC mPGK Promoter_F	CCACTGGTCAGGAAAGGAACACTAGTACG CGTTCTAGAGGGTAGG	Cloning
#7378	SLIC SV40polyA_R	CAGACACCCAGGATAAGTGCACTAGTTAAG ATACATTGATGAGTTTGGACAA	Cloning
#7379	SLIC CMV_F	CCACTGGTCAGGAAAGGAACACTAGTACG CGTTGACATTGATTATT	Cloning
#7388	Hipp11_F2	GCGGTCTTCATGTTCCACCCCAC	Genotyping
#7399	mPGK_prom_rev	CATCTGCACGAGACTAGTGAGACGT	Genotyping
#7418	CMV_prom_rev	GGAAAGTCCCGTTGATTTTGGTGCC	Genotyping
#7389	Hipp11_R3	TCAGGCTGCTGGAGAGTTGCACTCG	Genotyping
#7398	SV40_p(A)_fwd	TTCACTGCATTCTAGTTGTGGTTTGTCC	Genotyping
#7414	eGFP_MID_fwd	ACCCTGAAGTTCATCTGCACCA	Genotyping
#7413	eGFP_MID_rev	CGCTCACCTTCTTCTCCTGGAA	Genotyping

#7390	Hipp11_F4	TCAGGGCAGTCTGGTACTTCCAAG	Genotyping
#7391	Hipp11_R4	CCATCTCTCTAGCGTGTCTATACAC	Genotyping
#7557	eGFP_qPCR_fwd_1	GGGATCACTCTCGGCATGG	RT-qPCR
#7558	MicroID_qPCR_rev_1	AGCTCACGTTCCACTCCTTC	RT-qPCR
#7137	Gapdh_fwd_RT-qPCR	TGACCTCAACTACATGGTCTACA	RT-qPCR
#7138	Gapdh_rev_RT-qPCR	CTTCCCATTCTCGGCCTTG	RT-qPCR
