# Supplementary material for: Expression of transgenic biotin ligases in inducible neuronal murine cell lines by integration into the mHipp11 gene locus
Source: PLoS One. 2025 Mar 4;20(3):e0315806. doi: 10.1371/journal.pone.0315806 (PMC11878913; doi:10.1371/journal.pone.0315806)
Supplement: S2 Table — (PDF) [file pone.0315806.s008.pdf]

S2 Table. Antibodies used in this study.

Antibody/ Conjugate	Dilution	Company	Used for
AF594-Streptavidin	1:800	Thermo-Fisher (S11227)	Immunofluorescence
AF647-Streptavidin	1:500	Thermo-Fisher (S21374)	Immunofluorescence
HA	1:2000	Biozol (BLD-901513)	Immunofluorescence
GFP	1:1000	Roche (11814460001)	Western Blot
MAP2	1:10000	Biolegend (822501)	Immunofluorescence
NF200	1:100	Sigma (N0142)	Immunofluorescence
Oct3/4	1:200	Santa Cruz (sc-5279)	Immunofluorescence
Streptavidin-HRP	0.5 µg/ mL	Thermo-Fisher (S911)	Western Blot
Tubulin	1:5000	Sigma-Aldrich (T6199)	Western Blot
goat a mouse-AF488	1:1000	Invitrogen (A11001)	Immunofluorescence
goat a chicken-AF555	1:1000	Invitrogen (A21437)	Immunofluorescence
goat a mouse-AF555	1:1000	Invitrogen (A21422)	Immunofluorescence
goat a chicken-AF647	1:1000	Invitrogen (A21449)	Immunofluorescence
