# Supplementary material for: Expression of transgenic biotin ligases in inducible neuronal murine cell lines by integration into the mHipp11 gene locus
Source: PLoS One. 2025 Mar 4;20(3):e0315806. doi: 10.1371/journal.pone.0315806 (PMC11878913; doi:10.1371/journal.pone.0315806)
Supplement: Attachment — Submitted filename: Response_to_Reviewers_v2.1.docx [file pone.0315806.s009.docx]

Biochemistry I

Dr. Ralf-Peter Jansen

telephone +49 7071 29-72453

telefax +49 7071 29-5605

ralf.jansen@uni-tuebingen.de

http://www.ifib.uni-tuebingen.de

University of Tuebingen · IFIB · Morgenstelle 34· 72076 Tuebingen

Tuebingen, Oct 17, 2024

Dear Reviewers,

Please find uploaded the revised version of our manuscript " Expression of transgenic biotin ligases in inducible neuronal murine cell lines by integration into the mHipp11 gene locus" for publication in *PLoS One*.

First and foremost, we would like to thank both of you for the work and time you spent on assessing the initial manuscript. You will find our responses to your remarks below.

**Reviewer 1**

*Please identify the clones used for subsequent experiments and please label them accordingly in both the figures, figure legends and the text. As of current, I can only see Clone 11 and Clone 15 data mentioned in Figure 2B. I request the authors to identify to the clones used in the subsequent Figures 2C, 3, 4.*

We have added the requested information to all figure legends. In addition, we have specified the clones used in the main text on page 12 and again on page 13, where we state that all following experiments were done using the homozygous clone 15 only.

*Please also comment on the transgene silencing effects comparing Clone 11 and Clone 15, ideally providing data, if available.*

We have not been able to do this comparison since we have not performed these experiments on the heterozygous clone 11. Our reasoning was that the homozygous clone 15 should lead to a stronger expression, which we were originally aiming for.

*Concern: The DAPI channel exposure for figure 2C (3h) is noticeably higher than the rest.*

Figure 2 has been exchanged against a new figure 2 with new data. Fig 2C has been replaced by a new panel (Fig 2A). In this panel, the DAPI signals do not suffer from the problem mentioned above.

**Reviewer 2**

**Major comments:**

*- The authors presented qPCR data of the MicroID expression in Figure 4B. However, the total protein level of MicroID fusion is still missing for mESC, Neuron Day 8, and Neuron Day 10 cells. Also, did the authors test the levels of MicroID longer than ten days to confirm (or not) that the silencing is not happening later?*

1. We have tried to detect the MicroID protein by Western blot in whole cell lysates (WCLs) using its HA or GFP tags and different antibodies. Since we failed to obtain convincing MicroID fusion protein signals in these experiments in WCLs of mESC-derived neurons (but detected them in WCLs of mESCs), we performed GFP pulldown experiments from these lysates, followed by detection of the fusion protein by Western blot. Although the fusion protein could be detected after enrichment by the pulldown, the resulting Western blot images are of too low quality for quantification and to be used for publication. For reference, we have added a figure for review only (see supplied Figure_S6_for_review_only). Since we have been able to detect the MicroID-containing fusion protein in situ using an antibody against the GFP part (see new Fig 3A), we are convinced that we can confirm its expression in mHipp11 mPGK MicroID mESC-derived neurons.

2. We have not looked for expression levels in cells that have been differentiated longer than 10 days. We have adapted the differentiation protocol by Ludwik et al., 2019 (10.1016/j.ymeth.2019.02.002) for 8 and 10 days of differentiation since this already results in a high percentage of mESC-derived neurons (so called “iNeurons”) that is required and sufficient for the BioID experiments we are planning. The differentiation is also evident from the new S3 figure (NF200 and MAP2 staining). Cells at day 8 and 10 do not show any difference in staining or percentage of cells expressing these markers.

*- Similarly, it will be critical to evaluate the levels and subcellular localization of MicroID in mESC and neurons. This will help confirm the biotin labeling as well. Showing GFP images in live cells might show unwanted autofluorescence and background. Immunostaining the GFP fusion can help enhance the specific signal and lessen the non-specific background, especially in a non-GFP channel.*

We would like to thank the reviewer for this suggestion. We have performed in situ immunofluorescence experiments (a combination of anti-GFP and streptavidin-based biotin detection) that are now part of overhauled versions of figures 1 and 3 (each containing a new panel A). These experiments demonstrate that the MicroID fusion protein can be detected in the nucleus of mESCs and differentiated neurons (at day 10) and that a strong biotin signal can also be detected that largely overlaps with the nuclear signal of the MicroID fusion protein. We have thus deleted the live imaging on mESC-derived neurons but kept live imaging data of MicroID-GFP expressed in mESCs of different mHipp11 clones and mESCs after random integration. These are now part of the supplementary information (S2 Fig).

*- The authors will have to validate the pluripotency markers of the clone(s) used in Figure 3. After differentiation, neuronal markers have to be used to validate the derived neurons.*

We have validated the identity of mESCs and derived neurons with *in-situ* immunofluorescence detection of Oct3/4, NF200, and MAP2. This validation is found in the new supplementary figure S3 Fig and mentioned in the main text on page 13.

*- In Figure 3C, right panel, the images in red are not convincing. They will need to be shown with higher resolution, ideally co-stained with the MicroID fusion protein.*

Figure panel 3C has been deleted and replaced by a new figure panel (Fig 3A, see above). We believe that this panel now sufficiently demonstrates MicroID-dependent biotinylation in the nucleus of differentiated cells.

*- In Figure 3A, the authors stated that the wild-type (WT) cells show no nuclear staining. Fig 3A lacks DNA staining to be able to conclude with confidence the exact subcellular localization. Also, WT cells already display GFP-positive accumulation likely in the cell body. These issues will have to be addressed*.

Figure 3, panel A has been replaced by a new panel (Fig 3A; see above). We have replaced live imaging of GFP with anti-GFP immunostaining, and included streptavidin-AlexaFluor647 and DAPI staining. The resulting images more convincingly demonstrate the expression and localization of the MicroID-GFP fusion protein compared to wildtype cells.

*- Conclusions in lines 334–342: The authors will have to re-formulate these conclusions since we still see a very faint biotinylation effect coming from potential active biotin ligase in + biotin conditions in random genomic integration for mPGK MicroID and CMV TurboID (Fig4C)*.

We thank the reviewer again for this notification. We have adapted the main text accordingly, now replacing the statement that “no activity” was detected in cells with randomly integrated and CMV- or mPGK-driven MicroID by “far less activity”. Furthermore, we have replaced the final statement of this chapter that the biotin ligase is “only made and active” upon mHipp1 integration by a statement expressing that the MicroID is “more active in mESC-derived neurons when expressed from the mHipp11 safe-harbor locus compared to the version that is randomly integrated into the genome”.

**Minor Comments:**

*- We do not see in the result section which clone(s) the authors followed up with in Figure 3*.

As stated above in our response to reviewer 1, we have now specified in the figure legends (and the main text) what clones are used to obtain the results.

*- The authors will have to perform statistical analysis for fig4B and quantify the levels of AF594 for fig3C (or the updated fig3C)*.

We have performed an unpaired student’s t-test on the qRT-PCR data and added the results in Fig 4B. The previous figure Fig 3C has been deleted and replaced by the new Fig 3A, showing convincing co-staining of nuclear MicroID and biotinylation signals (see or comment above).

Sincerely,

Ralf-Peter Jansen
